# Towards automated planning for unsealed source therapy

**DOI:** 10.1120/jacmp.v13i4.3789

**Published:** 2012-07-05

**Authors:** Eduard Schreibmann, Tim Fox

**Affiliations:** ^1^ Department of Radiation Oncology and Winship Cancer Institute of Emory University Atlanta Georgia

**Keywords:** SPECT, unsealed sources, segmentation, registration, dose calculation, planning

## Abstract

The purpose of this study was to develop and validate a technique for unsealed source radiotherapy planning that combines the segmentation and registration tasks of single‐photon emission tomography (SPECT) and computed tomography (CT) datasets. The segmentation task is automated by an atlas registration approach that takes advantage of a hybrid scheme using a diffeomorphic demons algorithm to warp a standard template to the patient's CT. To overcome the lack of common anatomical features between the CT and SPECT datasets, registration is achieved through a narrow band approach that matches liver contours in the CT with the gradients of the SPECT dataset. Deposited dose is then computed from the SPECT dataset using a convolution operation with tracer‐specific deposition kernels. Automatic segmentation showed good agreement with manual contouring, measured using the dice similarity coefficient and ranging from 0.72 to 0.87 for the liver, 0.47 to 0.93 for the kidneys, and 0.74 to 0.83 for the spinal cord. The narrow band registration achieved variations of less 0.5 mm translation and 1° rotation, as measured with convergence analysis. With the proposed combined segmentation–registration technique, the uncertainty of soft‐tissue target localization is greatly reduced, ensuring accurate therapy planning.

PACS number: 87.55.de, 87.55.kd

## I. INTRODUCTION

The trend of more personalized therapy is driving clinical use of molecular imaging for assessment of therapeutic targets and identification of resistance factors to match therapy to tumor biology. Imaging can capture heterogeneity of target expression and measure the effect of radionuclide therapy on the target (i.e., receptor antagonism or change in target expression). However, personalized absorbed dose estimates of targeted radiotherapy are inaccurate at this time, but are essential to establish fundamental dose‐response relationships for efficacy and toxicity. Development of patient‐specific dosimetry for administered radionuclides, combined with molecular imaging, is important for better understanding of tumor response and normal‐tissue toxicity for individual patients.

Integration of functional single‐photon emission computed tomography (SPECT) imaging data into radiation dose calculations has drawn the interest of many researchers because of its compelling advantages in quantifying absorbed dose delivered to tumors and normal tissue for targeted radiotherapy.^(^ ^1^ ^–^ ^4^ ^)^ One of the most common applications is to treat hepatic malignancies by radioembolization with microspheres containing yttrium‐90 (${\;}^{90}Y$) delivered via the hepatic artery.^(^ ^5^ ^–^ ^8^ ^)^ In this approach, patient‐specific data from computed tomography (CT) or magnetic resonance imaging (MRI) provides an anatomical model with resolutions on the order of 1 mm. Similarly, SPECT provides three‐dimensional (3D) representations of activity distributions within patients, with typical resolutions of approximately 5–10 mm as a surrogate of dose distribution, to assess delivered dose and lung toxicity^(^ ^9^ ^–^ ^11^ ^)^ that significantly improves accuracy over standard approaches such as the MIRD formalism,^(^ ^12^ ^)^ or to guide the radiotherapy planning to spare critical tissue.^(^ ^13^ ^,^ ^14^ ^)^ Despite the extensive amount of work already done, many obstacles remain. Monte Carlo and dose convolution methods for calculating the absorbed dose have improved;^(^ ^15^ ^)^ however, image registration and anatomical segmentation methods require significant user interaction,^(^ ^2^ ^,^ ^3^ ^)^ which limit the clinical usefulness of the system.

Segmentation is the most time‐consuming step of the planning process. Methods based on thresholding or region growing are widely used, but are impractical when applied to soft‐tissue organs of similar intensities as only voxel intensities in the input images are used as guidance. When added to the segmentation process, prior knowledge of the shape and size of the organs to be segmented has increased accuracy,^(^ ^16^ ^–^ ^18^ ^)^ with atlas‐based–segmentation algorithms representing the most advanced approach to guide the segmentation process. The method relies on the existence of a mapping between a reference image volume (called atlas) in which structures of interest have been carefully segmented, and the image to be segmented (called subject). A point‐to‐point mapping between the two is obtained by a deformable image registration and is used to warp structures from the atlas onto the subject dataset. With this approach, the segmentation problem is reduced to a registration problem that tries to capture and compensate for interpatient anatomical variability.

Because the deformable registration can interpolate in regions of low contrast from neighboring anatomical features, the method effectively contours structures that do not have a clear border.^(^ ^18^ ^)^ This practical method was recently adapted in radiotherapy for CT datasets of the brain^(^ ^19^ ^)^ or head and neck region using automated methods such as B‐Spline,^(^ ^16^ ^,^ ^17^ ^)^ demons,^(^ ^18^ ^,^ ^20^ ^,^ ^21^ ^)^ or using a multimodality metric.^(^ ^22^ ^)^ The method was recently extended for atlas segmentation of breast cases^(^ ^23^ ^)^ but, to the best of our knowledge, this approach was not extended to segmentation of abdominal or thoracic datasets, because differences in patient size, organ location, or shape are significant and cannot be easily captured by current approaches.

Due to the low tissue contrast in the CT dataset and significantly different information in the SPECT dataset, automatic registration methods are unreliable and manual matching is still the norm. Direct application of multimodal matching methods on our SPECT‐CT datasets often failed, as the liver was the only structure visible in the SPECT image. Additionally, standard multimodality registration approaches assume a structure is represented by voxels of constant intensity, and large variations of the voxel intensities are observed in the liver for the SPECT images. Manual matching based on external markers^(^ ^24^ ^)^ neglect internal liver motion. Semi‐automated or automated registration setups have been reported that to some extent employ user guidance to match the two datasets. A semi‐automated method was introduced by Sarfaraz et al.^(^ ^10^ ^)^ that matches liver segmentations obtained from SPECT and CT datasets. Although the CT is segmented manually, the SPECT mask is obtained with an iterative threshold search that aims to match the volume of the CT segmentation. Tang et al.^(^ ^25^ ^)^ reported a voxel‐based approach using the Mattes formulation as metric function, and investigated the influence of number of bins and samples on optimizer convergence behavior. We reproduced their setup, but found the configuration produced inconsistent results on some datasets.

Our goal was to improve the registration and segmentation processes for treatment planning incorporating SPECT datasets by developing algorithms customized to the specifics of SPECT‐CT imaging datasets. The approach combines information from the segmentation and registration procedures into a common robust approach that simplifies the task. Tedious manual segmentation is replaced with structure identification through atlas registration that relies on an improved demons algorithm in which iterations are updated in a diffeomorphic space to account for large interpatient variations. A rigid registration method based on a hybrid approach combining advantages of the contour and voxel‐based approaches is introduced to accurately register anatomical and functional imaging data of the abdominal anatomy. In particular, we present our experience with selection of model parameters, optimization algorithm, and validation of the combined registration and segmentation technique.

## II. MATERIALS AND METHODS

**Figure 1:**
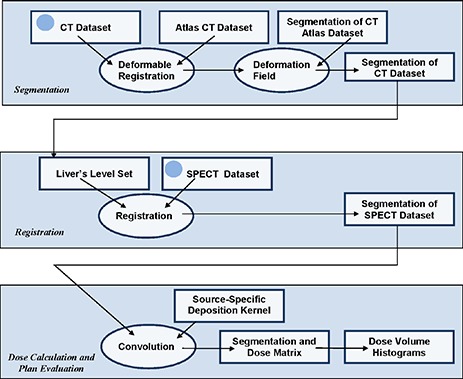
Flowchart of the proposed treatment planning procedure. Rectangles are data, ellipses are procedures, and arrows indicate data flow. Inputs are patient‐specific CT and SPECT data and are marked with blue circles. Nonpatient‐specific data input include the atlas CT, its segmentation, and the radionuclide‐specific deposition kernels. Output for each procedure is shown in the right lower corner.

A schema of the overall implementation of unsealed source treatment planning is presented in Fig. 1. It details the input and technical modules used to simplify the planning procedures in our approach. An atlas dataset is matched to the patient's CT dataset using deformable registration to produce a segmentation of critical organs (see Section II. B below). A narrow band of the liver segmentation was further used to guide registration, as described in Section II. C. Dose and plan evaluation measures were calculated by convolving the SPECT distribution with specific dose deposition kernels (Section II. D). All algorithms presented in the following were developed using the Insight Toolkit (ITK)^(^ ^26^ ^)^ library that implements a set of standard imaging filters as aids for developing customized algorithms.

### A. Datasets

Five patients received dual SPECT and CT scans following the routine clinical procedure. SPECT images were acquired on an ADAC SPECT system on a 360° orbit in step‐and‐shot mode, with a low energy‐high resolution (LEHR) collimator collimation for Tc‐99m, in using a 360° circular orbit in step‐and‐shoot mode, with an angular step of 7.05° and with an energy window of 126 to 155 keV (140 keV photopeak ±10%). The SPECT images were reconstructed using filtered backprojection with a Butterworth convolution kernel, and corrected for attenuation. Reconstructed SPECT volumes consisted of slices of 128× 128 size with cubic voxels of 3.2 to 4.6 mm size. The CT datasets were acquired on a LightSpeed RT 16 scanner (GE Healthcare, Waukesha, WI), in slices of 512× 512 voxels of pixel spacing 0.68 mm (35 cm FOV) with a slice thickness of 2.5 mm. The CT parameters used for imaging an exposure of 140 KVp and 210 mAs, and voxel sizes were 0.71, 0.71, and 2.50 mm.

### B. Atlas segmentation

**Figure 2:**
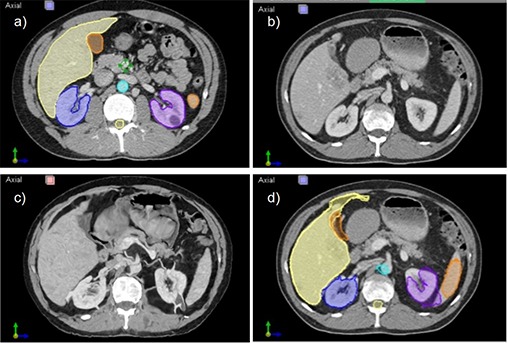
Concept of atlas based segmentation. The template CT dataset with the structures carefully segmented is shown in (a). The aim is to adapt the template dataset to the patient's CT (b) through deformable registration to reproduce organ boundaries. The warped template is shown in (c) and should ideally match (b) in major organs location and shape, with the unusual HU warping outside critical organs normal for interpatient registrations. The atlas segmentation as warped with the deformable registration result to segment the patient's CT is shown in (d).

The atlas segmentation approach (Fig. 2) starts with a template CT of a representative or average patient in which the clinical structures have been delineated by an expert, also called atlas. The template is then matched to the new patient image (called subject dataset) through deformable registration that deduces a one‐to‐one correspondence between the two CT datasets. The expert segmentation is then warped with the deformation field resulting from the registration to produce an automated segmentation of the subject dataset.

Accuracy depends critically on the deformable registration algorithm's ability to find the mappings that accurately match the anatomy between the atlas and subject. The atlas segmentation procedure has been proposed previously in the context of segmenting brain MRI datasets,^(^ ^27^ ^,^ ^28^ ^)^ where structures are clearly delineated. When attempting to extend the method to abdominal anatomy, much larger variability between subjects is a problem, compared to head and neck datasets. An algorithm's ability to cope with large differences customized for CT datasets and with running speeds of a few minutes were our primary criteria in selecting a deformable registration model for the abdominal atlas segmentation procedure.

We selected a deformable registration algorithm that extends the popular demons formulation. The demons algorithm warps the input images according to local characteristics with forces inspired from the optical flow equations. The method was improved by Vercauteren et al.,^(^ ^29^ ^)^ who used statistics on diffeomorphisms that rely on the computationally heavy solution of a partial differential equation. The selected implementation is an efficient nonparametric diffeomorphic image registration algorithm that uses an exponential update of the deformation field where significant accuracy improvement over the classical demons formulation is obtained, based on the algorithm's ability to cope with large deformations between patients.

As settings for the deformable algorithm, we used a step size of 2, a sigma value for the deformation field of 1, and 0.2 for the update field. For preprocessing, both the template and the patient datasets were smoothed using an edge‐preserving gradient anisotropic diffusion filter implementing the classical Perona‐Malik approach.^(^ ^30^ ^)^ A histogram filter was used before the registration to compensate for differences in HU calibration should the images come from different scanners. To speed up the registration and decrease the chance of it being trapped in a local minima, we used a multiresolution approach where images were registered first at a coarse level and then the result was used to initialize a full resolution registration. Our setup involved a 3‐stage multiresolution approach with 50, 40, and 30 iterations for each resolution.

Once the deformation field between the atlas and subject images was found, it was used to warp the segmentations. Binary images of each structure segmentation were warped with the deformation field, resulting in automated segmentation of the subject images.

### C. Narrow band registration

Rigid registration is a well‐established procedure that corrects for differences in scanner coordinate systems, patient posture, or anatomical changes between two image sets, by matching common features present in both datasets. For most applications in radiotherapy, such common features are easily identifiable in the form of anatomical features visible in both images. For matching functional SPECT to anatomical CT datasets, there is little (if any) common information in the image datasets. The nonuniform tracer deposition mainly within the liver is the only information visible in the SPECT dataset. In contrast, the CT dataset contains complete anatomical information, with organs represented by voxels of uniform intensities. This is obviously a multimodality registration, with assumption made in the standard mutual information (MI) metric not satisfied by the nonuniform intensities in the SPECT dataset. The liver shape is the only piece of information that is common in both datasets.

To automate the registration process based on specifics of the CT and SPECT datasets, we propose a registration method based on the narrow band approach.^(^ ^31^ ^,^ ^32^ ^)^ The key piece is usage of a narrow band to connect a liver segmentation in the CT dataset to the corresponding gradients of voxel activity in the SPECT images. The aim is to disregard interior liver voxels that are too nonuniform in intensities to allow reliable use of classical MI setups. The narrow band — a signed distance around an object of interest such as the liver — is used as optimization criteria to constrain the registration to relevant features without relying on the user's segmentation accuracy. As compared to pure intensity‐based registration metrics, the narrow band approach ignores irrelevant regions such as background and noise, as the metric is computed only on the pixels within the signed distance. However, the method is not as sensitive to segmentation as pure edge feature‐based methods, because the signed distance represents a transition zone around the edges that compensates for inaccuracies in the initial segmentation. A narrow band representation of liver segmentation is presented in Section III. B and the approach is described in detail by Tang et al.^(^ ^25^ ^)^ For the SPECT‐CT registration, the module was designed to fit the liver segmentation with the steepest gradient in the SPECT dataset. As settings for this task, we used a narrow band width of 10 mm and a regular step gradient optimizer with a maximum step of 2.

In one sense, this approach extends to the work of Sarfaraz et al.^(^ ^33^ ^)^ in matching borders rather than intensities during registration. In the proposed method, the registration itself: a) is completely automated, as it does not require mask of the liver in both datasets; b) increases accuracy, as it matches a segmentation to the gradients directly, without the use of user‐delineated masks in both images; c) increases speed, as the normal cross‐correlation (NCC) metric permits use of fast gradient‐based algorithms rather than the time–consuming simulated annealing optimization algorithm; and d) does not critically depend on the CT‐based segmentation accuracy, as narrow band itself defines an error margin around the initial segmentation.

### D. Dose calculation

Dose deposited per organ is implemented as a convolution operation of the SPECT activity concentration map with yttrium‐90 deposition kernels calculated using the EGS4 Monte Carlo simulations with an uncertainty below 1% to yield a three‐dimensional distribution of absorbed dose.^(^ ^10^ ^)^ The resulting dose distribution is then combined with the anatomical information to compute dose volume statistics for target and critical organs.

### E. Validation

Atlas‐based procedure accuracy was measured by comparing the automated results with the manual delineations using the Dice similarity coefficient (DSC) index,^(^ ^34^ ^)^ which is widely used in the evaluation of comparison studies. The coefficient is defined as the ration of the overlap of two structures over their union. The DSC conformality index is ideally 1 when the delineations to be compared overlap exactly, and is 0 when the delineations are completely mismatched. A value greater than 0.7 has been reported to indicate good segmentation performance.^(^ ^35^ ^)^

To measure registration accuracy, the true alignment is unknown and therefore we relied on a convergence analysis method to estimate accuracy. This method is based on repeating the optimization from different initial values. For a correctly configured registration algorithm, the final solution should be independent of the starting position. We restarted the registration 20 times from random initial displacements and inspected the transform parameters found in each registration attempt. The range of the final transform parameters is the measure of registration accuracy.

## III. RESULTS

### A. Atlas segmentation

**Figure 3:**
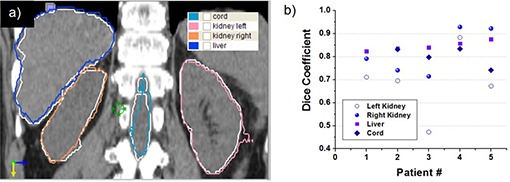
Sample result of atlas‐based segmentation (a) of the liver (blue), spinal cord (cyan), and kidneys (yellow and magenta). Manual segmentation of the same organs is shown in white for comparison. Dice coefficients (b) between atlas and manual delineations for all patients for critical organs. The ideal value is 1, denoting a perfect segmentation. A value greater than 0.7 denotes a good match.

A sample result of the deformable registration procedure is presented in Fig. 2. The upper row shows an axial slice through the atlas (a) and patient (b) datasets before registration. Natural anatomical differences between the two individuals are evident when comparing images. In (c) we show the result of deforming the atlas to the patient dataset, where the deformation field warped the atlas to match the patient dataset. The deformation field was further used to warp the segmentation between the two datasets as shown in (d). Little manual editing is necessary to correct regions where the atlas approach did not perform well, as show in the comparison between manual and atlas segmentation in Fig. 3(a). The Dice coefficient between the two segmentations for all structures for the five patients included in this study are graphed in Fig. 3(b), showing that with the exception of one kidney, all atlas segmentations were overlapped with the manual segmentation at a value of 0.7 or above.

**Figure 4:**
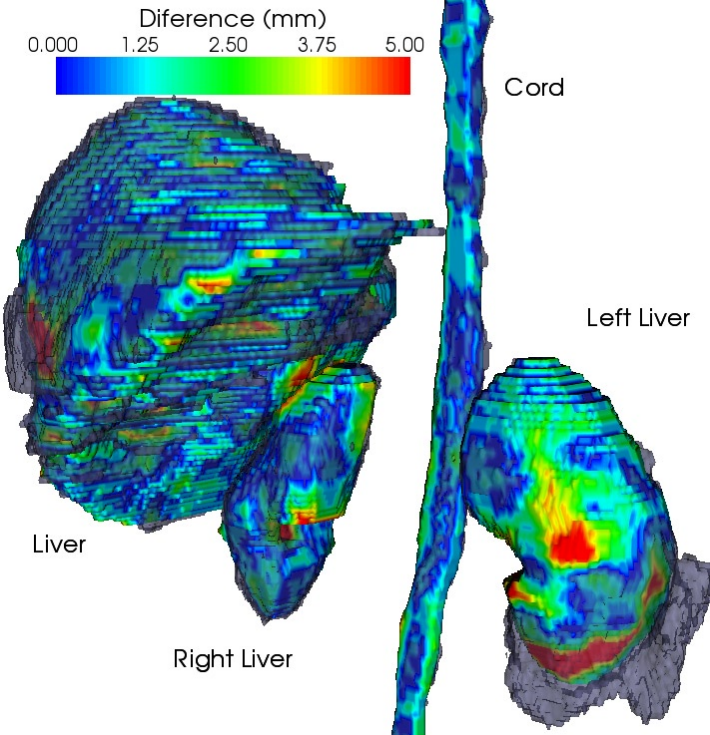
Three‐dimensional surface rendering of atlas‐based (gray) and manual (color‐coded) segmentations. The color on the surface represents the distances between the two segmentations, from 0 mm (blue) to 5 mm (red).

Results of the atlas‐based segmentation may be suboptimal due to different acquisition parameters or significantly different anatomy, as the selected deformable registration algorithm is single‐modality, assuming an organ is represented by the same intensity in both the template and patient datasets. We noticed that the selected deformable algorithm had problems, especially matching kidneys that have different shapes, are relatively small, and are surrounded by tissue of similar HU units. The liver is a large organ that significantly influences the cost function and, therefore, is preferentially matched with few distortions observed in our experiments. The spinal cord was easily matched as the surrounding high contrast provided by the bone protected it from artifacts and provided a clear mark easily matched by the registration. A 3D surface rendering of the suboptimal atlas segmentation result is presented in Fig. 4, where the automated segmentation of cord, kidneys, and liver is shown as a gray surface, and the manual as a color‐coded surface. The color on the surface represents the distance between the surface obtained by the two methods and ranges from 0 to 5 mm, with the gray surface representing the manual segmentation of the same organ.

### B. Narrow band registration

**Figure 5:**
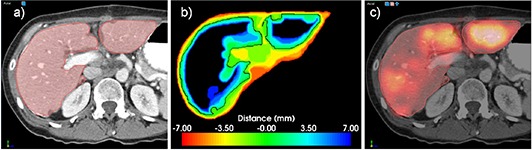
Example of a narrow band registration approach. The liver segmentation (a) is used to build a signed distance narrow band (b) that provides the link of the SPECT gradient with the liver segmentation used in registration. This allows usage of a simple NCC metric in the registration, since its minimum is achieved when the SPECT gradient matches the zero of the narrow band. A typical result is shown in (c), where registration clearly matched SPECT gradients with the liver contour. Overall the method yields reproducible results within 1 mm (shown in Fig. 6).

The main focus of our registration procedure is to match the gradient of the SPECT intensities to the liver segmentation obtained from the CT dataset by using a narrow band that compensates for segmentation inaccuracies and noise in the SPECT dataset. An example of a typical narrow band of liver segmentation is given in Fig. 5. The narrow band represented in (b) is an image in which voxel intensities represent distance from the initial segmentation presented in (a) as a black line. In the narrow band visualization, voxels are from blue to red according to the distance from the initial segmentation. The narrow band is then used to match to the SPECT image using a simple voxel‐based metric. Such a registration result is presented in the blended color fusion of (c), where the liver segmentation is a red contour and the SPECT intensity is displayed using a hot body lookup table. Indeed, the match is such that the SPECT gradient conforms to the liver segmentation. However, a manual match wouldn't be possible because a SPECT gradient is hard to detect by eye alone and is sensitive to window level settings. Registration resulting from our approach for this case is presented in Fig. 5(c). The registration matched the borders of the SPECT activity that appears as a red‐to‐yellow overlay in the CT dataset.

**Figure 6:**
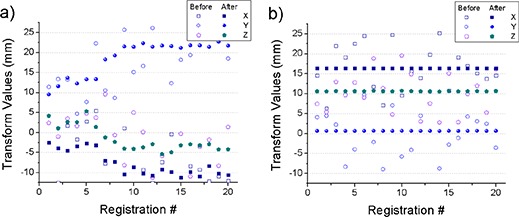
Convergence analysis for mutual information (a) and narrow band (b) registration method. Registration was started 20 times from random initial displacements. The graphs show initial and final displacements on the x‐, y‐, and z‐axis. For the mutual information setup, results were inconsistent. The level set‐based registration always converged to the same solution, achieving submillimeter accuracy.

Registration accuracy for our narrow band approach was assessed using convergence analysis and is presented in Fig. 6. The registration was repeated 20 times from random initial positions varying a few centimeters on the x‐, y‐, and z‐axes. The final solution was almost independent of the initial position, with the final solution varying less than 1 mm, as seen in Fig. 6(b), where after‐registration translation values are consistent. For comparison, the same analysis based on a classical approach using the Mattes formulation^(^ ^22^ ^)^ of the MI, coupled with the regular step optimizing the parameters of an affine transform, is presented in the left panel of Fig. 6, with a large variation in the final solutions. This is probably due to the global maxima being hidden by the levels of noise in metric function in the MI configuration for the levels of inhomogeneity in the SPECT‐CT images.

**Figure 7:**
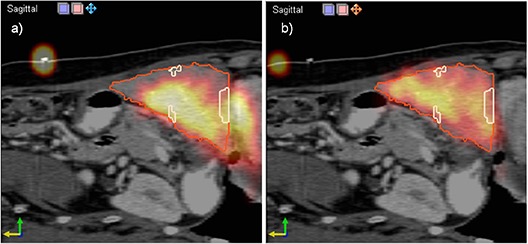
Comparison of registration methods using manual dual SPECT‐CT markers (a) and the narrow band approach (b). The largest error was observed in the manual match due to internal motion. The mutual information and narrow band registrations agree for this case.

One of the patients had dual SPECT‐CT markers that allowed comparison of the manual and automated registration methods. The registration based on manually matching the thoracic marker is presented in the left panel of Fig. 7. Although the three thoracic markers were carefully matched, internal liver SPECT distribution does not match to the CT dataset because some activity is reported in the heart and there is no activity for the CT‐delineated liver lesions (contoured in red). This was attributed to liver motion during respiration. The right panel shows the result of the narrow band registration, which has a better match as the activity is contained in within the liver.

### C. Dose calculation

**Figure 8:**
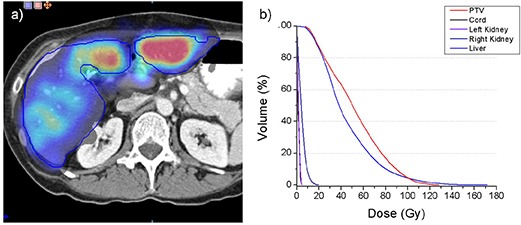
Dose evaluation tools show an axial display (a) and a dose volume histogram (b) of the dose distribution.

A display showing the dose distribution as a semitransparent overlay superimposed on the CT scan of a patient treated with radioactive microspheres is presented in Fig. 8. Dose evaluation tools show the distribution (a) and a dose volume histogram (b) of the calculated dose. Target (tumor and liver) dose ranges from 20 to 140 Gy. The maximum dose to the other critical structures is less than 20 Gy.

## IV. DISCUSSION

Our goal was to design an integrated image‐guided dosimetry planning system for predicting therapeutic response using targeted radiotherapy delivery to provide clinical decision support software for unsealed source therapy. In particular, we improved the registration and segmentation tasks by customizing general purpose registration algorithms to specifics of the CT and SPECT images for the special case of liver‐directed Y‐90 microspheres SIRT based on Tc‐99m MAA as a surrogate. First, choosing to use the atlas algorithms based on deformable registration, we segmented data more efficiently than in a manual approach, since the contours from a template can be warped to match the patient's anatomy (Fig. 2). As shown by our results (Figs. 2, 4, 5), the template segmentation can be successfully transferred to the patient dataset through deformable registration. The Dice coefficient comparison (Fig. 3(b)) confirms that the proposed automated method is close to the manual segmentation, but is significantly faster and requires less user interaction. The registration is based on a narrow band formalism (Fig. 5) to deal with the nonuniform intensity distribution in the SPECT images. For all cases, the convergence analysis (Fig. 6) measured an accuracy of 1 mm for the proposed registration method, and confirmed algorithm robustness. Dose‐volume analysis of structures is presented to the clinician for predicting the response to targeted radiotherapy using Y‐90 microspheres (Fig. 8). The plan could also be modified for the dose‐painting technique, to deliver a higher dose to the metabolically active portion of the tumor and a lower dose to the other abnormalities visualized within the liver where the signal is higher than the background but less than in the confirmed tumoral regions.

Validation of atlas‐based segmentation was achieved through comparison with manual delineation. The procedure achieved good accuracy as measured by the Dice coefficient, with some postprocessing needed clinically to locally correct output. The most common artifact was incomplete kidney segmentation, which can be corrected using either manual editing or an automated local refitting function. The SPECT‐CT narrow band registration was validated through convergence analysis, with measured accuracy less than 1 mm — a significant improvement over classical MI‐based approaches. The dose calculation model implemented through the image convolution algorithm does not constitute a novelty and, therefore, accuracy was not explicitly assessed.

To register the SPECT with CT images, theoretically, all we need to do is to use an optimization algorithm to minimize a MI metric. Such setups have been applied before to problems of CT‐MRI registration and are in clinical practice. For SPECT and CT datasets, due to their fundamentally different nature, the MI metric is noisy and the system finds different solutions if initialized differently. This is attributed to the wide variety of possible pixel intensities within an organ in the SPECT dataset. Reported narrow band formalism was devised to improve the convergence calculation by replacing complex MI calculations with simpler considerations of the voxel‐based metric. The approach improved accuracy over classical MI approaches, and is significantly faster and easier to use than manual^(^ ^24^ ^)^ or surface‐matching approaches.^(^ ^33^ ^)^ Although data for SPECT‐CT registration is presented, the approach is also applicable to MRI‐SPECT registrations.

Hybrid SPECT‐CT scanners are commercially available for clinical applications with the advantage that by acquiring both sets of images on the same scanner, the hybrid setup provides better anatomic localization of lesions than standard nuclear medicine images, but involve significantly higher costs and hardware complexity. Although this development alleviates the need for a rigid image procedure to correct for differences in scanner coordinates, as both datasets are acquired on the same device, the significantly larger acquisition time leads to the SPECT tracer being smeared by the respiratory motion^(^ ^36^ ^,^ ^37^ ^)^ resulting in an averaging of detected signal. The much faster CT acquisition images the anatomy at a narrow respiratory phase. This discrepancy cannot be taken into account by hardware‐only solutions. The proposed automated rigid registration can be employed to further improve accuracy by compensating for respiratory motion through a rigid shift. The proposed registration procedure also provides a low‐cost alternative to the expensive and complex hardware approach required in hybrid CT‐SPECT scanners.

In this work, Tc‐99m MAA is employed as an imaging surrogate for calculation of dose distribution within the organs to be spared as it provides higher resolution and is currently more quantitatively accurate, compared to Y‐90 imaging. The method provides merely an estimate of the delivered dose, as Tc‐99m MAA is known to breakdown biologically following administration, with both free Tc‐99m and Tc‐99mO4 migrating to other organs, whereas Y‐90 microspheres do not. This makes MAA a suboptimal surrogate for treatment planning, overestimating the dose delivered to critical organs. In addition, the Y‐90 microsphere delivery is often altered with respect to that for the MAA based on overall treatment planning for this procedure, and thus the two biodistributions may not match.

We considered the special case of Y‐90 microspheres that is the simplest of all unsealed radionuclide therapies to model. For this permanent implant treatment methodology, a single time point SPECT can potentially accurately model the biodistribution that is confined to two large organs, liver and lung, with essentially instantaneous uptake and clearance by physical decay only. However, most applications of internally administered therapeutic radionuclides are systemic, with biodistribution into a number of organs, and biologic uptakes and clearances that require multiple time point scanning to accurately model the biodistribution into multiple organs. As continuation of this work, we plan to extend the proposed methodology to multiple time point SPECT to improve tracer deposition modeling for treatment planning.

## V. CONCLUSIONS

We improved the segmentation, registration, and dose calculation steps for unsealed source therapy treatment planning of liver‐directed Y‐90 microspheres SIRT based on Tc‐99m MAA as a surrogate by using customized image registration setups. Our study indicates that this atlas segmentation approach produces results that need little, if any, manual refining, significantly reducing delineation time. Validation in patient studies of the customized image matching approach has indicated that the registration is reliable and thus provides a valuable tool for integrating SPECT information into radiation therapy treatment planning. This might have a significant practical implication with the prevalence of unsealed source therapy in clinical practice
